# Stone in the Urinary Bladder—Report of Case

**Published:** 1899-01

**Authors:** A. N. Denton

**Affiliations:** Austin, Texas


### Stone in the Urinary Bladder—Report of Case.

BY A. N. DENTON, M. D., AUSTIN, TEXAS.

Read at Austin District Medical Society, December 22, 1898.

The salts held in solution in normal urine are liable at all times to deposit in the form of calculi, either in the kidneys of bladder, or they may be imprisoned at any point along the course of the urinary tract. These calculi are, in a majority of cases, composed of urates, but cases in which the stone is composed of calcium oxalate, and possibly phosphates, are not by any means rare. I believe vesical calculi generally have their beginning in the kidney, and having traversed the ureter and reached the bladder, gradually become larger by new accretions from the urine.

Phosphatic calculi may be due to chronic cystitis, which favors and maintains alkalinity of the urine. Uric acid calculi are usually smooth, round or oval, and rather hard, but easily broken, whilst the calculi composed of oxalate of lime have a rough and uneven surface, although the general contour may be, and usually is, regularly oval, or round. These physical properties of the oxalate of lime calculi have suggested the name, "mulberry calculus." These are very hard, and I believe are formed more slowly than the calculi composed of urates.

Calculi composed of the earthy phosphates, that is, of lime, magnesia, and ammonia (triple phosphates), are of lower specific gravity than other varieties. They are white, soft and smooth, and hence cause less pain and vesical irritation than the "mulberry calculus," The composition of a stone, however, is not always uniform. It may have a uric acid nucleus, and then a layer of phosphates, etc. These alternating layers of uric acid and phosphates are doubtless due to varying conditions of the urine.

The causes of urinary calculi are quite varied. In a general sense, anything that favors urinary deposits may cause calculi, either of the kidney or bladder. Hence, indigestion, excess of solids and nitrogenous diet and deficient exercise may contribute to the formation of calculi.

In children, the uric acid stone is much more common than other varieties, whilst the triple phosphate stones are more common in the aged. Especially is this true in old men with enlarged prostates and chronic cystitis. In females, stone is rare, for obvious reasons; and for reasons not so easily discoverable, it is also rare in the negro race.

The predisposing causes are numerous. The principal are rheumatism, gout, enlarged prostate, urethral stricture, and catarrhal inflammation of the kidneys or bladder. The symptoms are also numerous, but there are *none* that justify operative procedure except touching the stone with a sound in the bladder.

The symptoms which may lead the surgeon to suspect the presence of stone are (1st) frequency of micturition, especially in day time, the desire to empty the bladder being sudden and uncontrollable, and being aggravated by excessive (2nd) pain, which is sharp, severe, and of a burning character, at the conclusion of micturition, which is always suffered to the end of the penis. The pain is due to the contraction of the empty bladder upon the stone, and is more or less severe in proportion to the size of the stone and the roughness of its surface.

A calculus of small size of the "mulberry" character, will cause more pain than a large one with smooth surface.

The passage of blood may or may not be present. If passed, it is usually after excessive pain. Pus, or muco-pus, may also be passed.

The failure to detect the presence of the stone with a steel sound, is not always conclusive evidence of the non-existance of stone in the bladder. Nature may have, for its own protection, caused the stone to become encysted, or covered with a false membrane or new growth, in order to render the stone stationary, and thus save the bladder from the irritation caused by the movement of the stone.

I shall not weary you with a discussion of treatment for the purpose of preventing the formation of stone, which will of course vary with the condition of the patient and the urine. When a stone is once formed in the bladder, there are only two methods of procedure open to the surgeon, viz., lithotopaxy, or lithotrity, and lithotomy. The former operation should never be attempted unless the bladder is of sufficient capacity to conveniently hold six or eight ounces of liquid. 2nd, in order to success the stone should not be too hard or too large, and of course the urethra should be tolerant and penetrable by the necessary instruments.

It is not, however, my purpose to discuss all the necessary conditions to successful lithotrity, nor need I describe the operation. I only desire to say that the existing conditions in the case which I am about to report excluded the possibility of a successful lithotrity, and therefore lithotomy was the only remaining resource.

We all know that there are two lithotomy operations—the lateral or perineal operation, and the supra-pubic operation. The former operation is growing less popular, and I believe will be abandoned altogether at no very distant period; however, it is but just to say that many leading surgeons still prefer the perineal route to the bladder. Just why it should be preferred by any experienced surgeon, is difficult to say. At all events, I think, in the vast majority of cases, the supra-pubic operation is to be preferred, for several reasons. The former operation is quite inadmissible in cases where the stone is very large or very hard, or in case of multiple calculi, also in men with enlarged prostates, or in cases with very deep perineum or very narrow outlet. In all these conditions, the perineal operation should not be considered. But even in most, if not all other cases, I think the "high" operation, as it is sometimes called, is generally preferable to the other.

The operation for stone in the bladder is common enough, and of course there is nothing new in the case which I am going to report. I think, however, there are some practical inferences to be drawn from the case, which I desire to impress upon the members of the society.

In every case of stone, at least a week should be taken in which to prepare the patient for the operation. During this time the patient, as a rule, should be kept in bed, and the bladder should be washed out daily with a warm boracic acid solution, the bowels should be kept open, and on the night previous to the operation a saline purgative should be given, and on the morning of the operation an injection of warm water into the rectum should be administered; and a bath should also be given.

These preliminary measures were fully complied with in the case of J. N., male, aged 4 years, whose parents consulted me about September 20, 1898. The boy's parents gave a history of ill health for the past two years, the most prominent symptom being irritable bladder, with intense pain after micturition. The parents stated that the boy had not slept more than half an hour at once during the past six months, and as a natural consequence his nervous system was much disturbed. The urine was found to be abnormally acid, and a vesical calculus was at once suspected, which was confirmed by an examination with a steel sound.

An operation was proposed, and consented to by the parents, and was done on the morning of September 26, 1898, assisted by Drs. Bennett and Hudson of this city, and F. R. Martin, of Kyle. The supra-pubic operation was chosen, and in order to make the bladder more prominent in front, an attempt was made to distend it with a warm solution of boracic acid, which was only partially successful, as the organ was found to be so contracted as to render it impossible to inject into it more than two or two and one-half ounces of fluid. With a tape around the penis to prevent escape of the fluid, and with the patient anæsthetized, an incision was made in the median line, beginning at or very near the syphysis pubis and extending two and one-half inches in the direction of the umbilicus. Owing to the small size of the bladder and the imperfect distension, it was somewhat difficult to keep the peritoneum pushed up out of the way in reaching the bladder. It was at length reached, however, and hooked up with a tenaculum, and an attempt was made to drag it forward into the wound, which was only partially successful, owing to its lack of distension. Finding it impracticable to incise the organ with accuracy under existing conditions, at this stage of the operation the tape was removed from the penis, and a steel sound was passed into the bladder, and by this means the organ was pushed forward and incised, after first passing a strong silk ligature through it near the upper angle of the wound.

The left index finger was then passed into the bladder, and the stone felt, and was found to be partially incysted. Guided by the finger in the bladder, several futile attempts were made to grasp and remove the stone with forceps. Failing in this, it was finally dislodged from its capsule and removed with the finger.

After thoroughly washing away all fragments and detritus, the opening in the bladder was closed with cat gut sutures, and the external wound with silk sutures.

The patient reacted nicely, and in three hours passed his urine through the natural channel. The case seemed to do well for two days, with very little rise of temperature, and the wound seemed disposed to heal by first intention throughout its entire length. In the night of the 28th, the bladder, from neglect, became unusually distended, and in a fit of crying the urine was forced through the anterior wall of the bladder along the course of the incision, into the peri-vesical tissues, and was there confined, the skin and subjoined tissues having almost completely adhered. The patient continued to pass urine per urethra, and the leak in the bladder was not discovered until the evening of the 29th. Meantime pain was lighted up, and the temperature arose to 102½ degrees.

Several sutures were at once removed, and the wound was opened and drainage established; but in spite of these measures urine was infiltrated downward to the right side in the direction of Poupart's ligament, and an abscess was formed in that region, which was evacuated on the 30th, and afterwards kept carefully evacuated and cleansed.

I should have said, that as soon as the break in the anterior wall of the bladder was discovered, a small sized, soft rubber catheter was introduced, and from this time onward the bladder was kept drained, in order to give the wound in front a better chance to heal. The abscess cavity rapidly filled up, and in a week there was nothing left except an opening but little larger than a lead pencil leading down into the bladder, and all fever had subsided. In another week the opening into the bladder was closed, and the boy was again passing his urine through the natural channel. At the end of the fourth week, he was discharged cured, and returned to his home.

Now, let us examine, and, if practicable, point out the mistakes that were made in the case.

1st. The operation should not have been undertaken without having first adopted measures to push the bladder well to the front. As it was found impossible to distend the organ by the injection of fluids, the rectal air bag should have been resorted to.

2nd. The bladder should not have been closed with ordinary cat gut, as its holding qualities are too transient to give time sufficient for permanent closure of the vesical wall. Formalin cat gut is far more enduring, and should have been used.

With this precaution, the pus vesical abscess, and urinary fistula, may have been avoided, and a more satisfactory and quick recovery secured.

